# TCA cycle intermediates as an adjunct strategy for human iPSC-derived cardiomyocyte maturation

**DOI:** 10.1016/j.bbadva.2026.100183

**Published:** 2026-02-16

**Authors:** Keshav Narayan Alagarsamy, Emilee Bueckert, Mehak Gupta, Michel Aliani, Sanjiv Dhingra

**Affiliations:** a Institute of Cardiovascular Sciences, St. Boniface Hospital Albrechtsen Research Centre, Department of Physiology and Pathophysiology, Max Rady College of Medicine, Rady Faculty of Health Sciences, University of Manitoba, Winnipeg, Manitoba R2H 2A6, Canada; b Division of Neurodegenerative Disorders, St. Boniface General Hospital Albrechtsen Research Centre, University of Manitoba, Winnipeg, Canada

**Keywords:** iPSC-CM, Metabolism, Maturation, Metabolomics & TCA cycle

## Abstract

Human induced pluripotent stem cell-derived cardiomyocytes (iPSC-CM) are a valuable tool for modeling cardiac diseases, drug testing, and regenerative applications. However, their application is limited by the immature phenotype of iPSC-CM. During maturation from fetal to adult phenotype cardiomyocytes undergo a transition from glycolysis to oxidative phosphorylation for energy production, which is supported by efficient tricarboxylic acid (TCA) cycle activity. Our metabolomics data suggest that the level of intermediates of TCA cycle including succinate, malate, fumarate, and α-ketoglutarate was very low in iPSC-CM. Therefore, we investigated the effect of supplementation with these metabolites on the maturation of iPSC-CM. We cultured iPSC-CM in glucose (Glu), galactose (Gal), or galactose plus TCA cycle intermediates (Gal+TCA) and evaluated the incremental effect of TCA cycle intermediates supplementation relative to Glu and Gal. The treatment with these TCA cycle intermediates led to improved calcium handling and cellular morphology of iPSC-CM relative to Glu and Gal. Furthermore, the treatment with TCA cycle metabolites enhanced electrical activity, improved mitochondrial health, and the cells were shifting toward oxidative phosphorylation relative to Glu only. This shift in the energy metabolism was associated with an upregulation in the expression of cardiomyocyte maturation genes and downregulation in the expression of fetal genes in Gal + TCA group relative to Glu. Overall, the benefits of Gal+TCA supplementation were quite evident compared to Glu alone but generally modest relative to Gal supplementation, supporting that TCA cycle intermediates supplementation can be used as an adjunct strategy to promote iPSC-CM maturation.

## Highlights

• Targeted metabolomics demonstrate that TCA cycle intermediates play an important role the maturation of iPSC derived cardiomyocytes.
• Supplementation of succinate, malate, fumarate and α-ketoglutarate promotes metabolic and functional maturation of iPSC derived cardiomyocytes.

## 1 Introduction

Human-induced pluripotent stem cell-derived cardiomyocytes (iPSC-CM) have become indispensable tools in cardiovascular research, providing a human-specific platform to study cardiac disease, screen new drugs, and explore regenerative therapies [1]. Their patient-specific genetic profiles has proven especially valuable for modeling inherited cardiomyopathies, arrhythmogenic disorders, and heart failure in vitro, thereby advancing the understanding of disease mechanisms and enabling the development of targeted treatments [2,3]. In pharmacological research, iPSC-CM support high-throughput screening for cardiotoxicity and proarrhythmic risk, contributing to safer and more efficient drug discovery pipelines. Additionally, these cells are under active investigation for applications in regenerative medicine, including myocardial repair via cell transplantation and bioengineered heart tissues [4,5]. Despite these promising advances, iPSC-CM remain structurally and functionally immature compared to adult cardiomyocytes, limiting their capacity to fully replicate native cardiac physiology [6]. Overcoming this immaturity particularly in electrophysiology, contractility, and metabolism remains a critical challenge and a focal point of current efforts to enhance the clinical and translational relevance of iPSC-CM.

Metabolic maturation is a critical determinant of the functional competence of iPSC-CM, which typically exhibit an immature metabolic phenotype characterized by a reliance on glycolysis for ATP production. In contrast, adult cardiomyocytes generate energy predominantly through mitochondrial oxidative phosphorylation, supported by robust fatty acid oxidation and efficient tricarboxylic acid (TCA) cycle activity to sustain contractile function. This transition from glycolytic metabolism to an oxidative state is a hallmark of cardiomyocyte maturation and remains a major bottleneck in the development of physiologically relevant mature phenotype of iPSC-CM [[7], [8], [9]]. To address this, several strategies have been employed to stimulate metabolic maturation. The fatty acid supplementation has been shown to enhance mitochondrial respiration and upregulate the expression of oxidative enzymes [10,11]. In parallel, activation of key regulators of cell metabolism including PGC-1α [12], AMPK [13], and PPARδ [14] has been shown to promote mitochondrial biogenesis and oxidative metabolism, contributing to improved sarcomere structure and calcium handling. However, all of these strategies have not been able to generate mature phenotype of iPSC-CM. In the current study iPSC-CM were subjected to targeted metabolomic analysis with a focus on glycolytic and TCA cycle intermediates. The TCA cycle is central not only to cellular energy production but also to metabolic signaling and epigenetic regulation, both of which are critical in directing cell fate decision [15]. In the metabolomics study, we observed significantly diminished levels of TCA cycle intermediates in iPSC-CM. Therefore, we hypothesized that exogenous supplementation of specific TCA cycle intermediates may promote metabolic and functional maturation of iPSC-CM. This study systematically investigates the effects of these TCA cycle metabolites on the electrophysiological properties, mitochondrial metabolism, and structural maturation of iPSC-CM.

## 2 Materials and methods

### 2.1 iPSC generation and differentiation to iPSC-CM

The iPSC were generated by reprogramming peripheral blood mononuclear cells (PBMC) from healthy individuals using our previously described protocols [16,17]. The iPSC were characterized using pluripotency markers such as SOX2, NANOG, OCT4, TRA (1–81,1–60) and SSEA4. The iPSC were passaged every 4 days using accutase (Thermo Fisher) in the presence of 5 μM ROCK inhibitor (Tocris) to enhance cell attachment [[18], [19], [20]]. The cells were routinely split at a 1:12 ratio, and the culture medium was changed daily. Cardiomyocyte differentiation was carried out using our previously published protocol [19,[21], [22], [23], [24], [25]]. Briefly, the iPSC at >90 % confluency were cultured in CDM3 medium consisting of RPMI 1640 (Thermo Fisher), 500 μg/ml recombinant human albumin (Sigma-Aldrich), 213 μg/ml L-ascorbic acid 2-phosphate (Sigma-Aldrich), and 5 % Antibiotic-Antimycotic (Thermo Fisher). The differentiation was initiated with 4 μM CHIR99021 (Tocris) in CDM3 for 2 days, followed by 2 μM Wnt-C59 (Calbiochem) for 2 days. The cells were then maintained in CDM3 alone for 6 days, with media changes every other day. The spontaneous contractions typically appeared between days 7–10. For purification, the cells were cultured in glucose-free CDM3 supplemented with 4 mM L-lactic acid (Sigma-Aldrich) for 3 days. On day 15, iPSC-CM (>70 % purity) were dissociated using TrypLE 10X (Thermo Fisher) for 15–20 min and replated onto Geltrex-coated plates at 100,000 cells/cm² in RPMI 1640 with 20 % FBS and 10 μM ROCK inhibitor. After 24 hours, the medium was switched to RPMI with 2 % B27 and maintained for 2 additional days prior to experimentation.

### 2.2 Media preparation

To investigate the impact of different metabolic substrates on iPSC-CM, three distinct media formulations were prepared using a base medium consisting of RPMI 1640 supplemented with 2 % B27 (Thermo Fisher, 1X), lipid mixture 1 (Sigma Aldrich, 6 mL/L), MEM vitamin mixture (Thermo Fisher, 1X), and Antibiotic-Antimycotic (Thermo Fisher, 1X). a) The glucose-rich (Glu) medium was prepared by adding 500 μL of 1 M glucose to the base medium to achieve a final concentration of glucose to 10 mM. b) The galactose-rich (Gal) medium contained 5 mM galactose and 5 mM glucose, added directly to the base medium. c) The third formulation, Gal + TCA medium was prepared by supplementing the Gal medium with 0.025 mM each of freshly prepared TCA cycle intermediates (di-sodium DL-malate, alpha-ketoglutarate, sodium fumarate dibasic, and sodium succinate dibasic hexahydrate) (Sigma Aldrich). All media were freshly prepared, sterile-filtered, and used immediately in downstream applications.

### 2.3 Immunofluorescence

Immunofluorescence was used to characterize iPSC and iPSC-CM, and to assess structural changes following 7-day of iPSC-CM culture in different media. The cells were fixed with 4 % paraformaldehyde (Thermo Fisher) for 15 min at room temperature and washed twice with PBS. The blocking was performed for 60 min in PBS containing 5 % BSA and 0.3 % Triton X-100 (Sigma-Aldrich). Primary antibodies were applied in PBS with 3 % BSA and 0.3 % Triton X-100 and incubated overnight at 4°C. After three washes of 5-minute each with PBS containing 0.3 % BSA and 0.03 % Triton X-100, the cells were incubated with secondary antibodies for 60 min in the dark. Finally, the samples were mounted using ProLong Diamond Antifade Mountant with DAPI (Thermo Fisher) and dried overnight. The imaging was performed using a Nikon Ti-2E fluorescence microscope. The antibodies used are listed in (**Fig. S1**).

### 2.4 Targeted metabolomics

The targeted metabolomics was performed using GC–MS/MS by following our previously published protocol [26]. Briefly, for analysis, standard solutions of fumarate, malate, lactate, and α-ketoglutarate were prepared in methanol (1 mg/mL), while other metabolites were dissolved in water at the same concentration. A mixed standard cocktail was created and serially diluted in methanol to generate working standards. The derivatization was performed by adding 30 μL of BSTFA with 1 % TMCS to each sample and incubating at 37 °C for 30 min. After derivatization, 60 μL of each sample was transferred to micro-vial inserts and stored at −80 °C. For sample preparation, cells were washed with PBS and extracted using 500 μL of 75 % methanol:MTBE (9:1, v/v) containing 5 μg/mL of 1,2-¹³C₂ myristic acid. Following freeze–thaw lysis and centrifugation, supernatants were dried under nitrogen and stored at −80 °C. The GC–MS analysis was performed using an Agilent 7890B GC system coupled to a 7000D Triple Quadrupole MS using HP-5MS column. The temperature ranged from 60 °C to 300 °C, with helium as the carrier gas and nitrogen for collision. The data were analyzed using Mass Professional Profiler (v15.1). For the metabolite uptake experiment, iPSC-CM cultured in Gal or Gal + TCA conditions were normalized to cell number prior to comparisons.

### 2.5 Mitochondrial membrane potential

Mitochondria membrane potential (MMP; Ψm) of iPSC-CM was assessed using tetramethyl rhodamine ethyl ester (TMRE) assay. The iPSC-CM were plated at a density of 100,000 cells/cm^2^ and treated with different concentrations of TCA cycle intermediates (0.025, 0.05, 0.1, 0.25 & 0.5 mM) for 7 days. After the treatment cells were washed with PBS and treated with100 nM TMRE at 37°C with 5 % CO₂ for 30 min. The fluorescence intensity was measured using Cytation 5 (BioTek Instruments) with excitation and emission wavelengths of 549 nm and 575 nm, respectively. Each group included 7 biological replicates.

### 2.6 LDH assay

The cytotoxicity of metabolites toward iPSC-CM was assessed using lactate dehydrogenase (LDH) release assay, by following the manufacturer’s protocol (Thermo Fisher Scientific). The iPSC-CM were plated at a density of 100,000 cells/cm² and treated with increasing concentrations (0.025, 0.05, 0.1, 0.25, and 0.5 mM) of individual TCA cycle intermediates (succinate, fumarate, malate, and alpha-ketoglutarate) for 7 days. At the end of the treatment period, culture media were collected, and LDH release into the supernatant was measured as an indicator of membrane integrity and cytotoxicity. The absorbance was recorded at 492 nm using the Cytation 5 Imaging Multi-Mode Reader (BioTek). The LDH activity values were normalized to total cell number to account for any variations in seeding density or due to cell proliferation. Five to Six biological replicates were analyzed per condition to ensure statistical robustness.

### 2.7 Multielectrode array

The electrophysiology of iPSC-CM cultured in Glu, Gal, and Gal + TCA groups were assessed using the MEA System (Axion Biosystems). The Cytoview MEA plates were pre-coated with a 1:100 dilution of Geltrex in DMEM/F12, and iPSC-CM were seeded at density of 75,000 cells per well across 24-well plates. The field potential measurements were recorded on day 7 post-treatment using Axis Navigator 3.5.1 software. Following a 10-minute stabilization, spontaneous cardiac activity was recorded for 10 min at 37°C and 5 % CO2. The key parameters including Beat Amplitude, Excitation-Contraction Delay and Beat Period were analyzed, with 7–8 biological replicates per condition.

### 2.8 Calcium imaging

Calcium flux in iPSC-CM cultured in Glu, Gal, and Gal + TCA groups was assessed using 3 μM Fluo-4 AM dye (Thermo Fisher), following manufacturer's protocol. The cells were incubated with the dye in Live Cell Imaging Solution (Thermo Fisher) for 45 min at room temperature in the dark, washed with PBS, and then incubated for 10 min at 37 °C to allow de-esterification. The calcium transients were recorded as 30-second video loops at 10 frames per second using a Nikon Ti2 inverted fluorescence microscope. The data were collected from 7–10 regions of interest per video, analyzing more than 40 beats per condition. Analysis was performed using Clampfit 10.7.0.3 and Nikon AR software, with 6–7 biological replicates per group.

### 2.9 Quantitative PCR (qPCR) analysis

Total RNA was isolated from iPSC-CM in Glu, Gal, and Gal + TCA groups using the Aurum Total RNA Mini Kit (Bio-Rad) following manufacturer’s protocol. The RNA concentration and purity were measured with a NanoDrop spectrophotometer (Thermo Fisher Scientific). cDNA was synthesized using iScript gDNA Clear cDNA Synthesis Kit (Bio-Rad), and quantitative real-time PCR was performed using SYBR Green Master Mix (Bio-Rad) on the CFX384 Touch Real-Time PCR System. The gene expression levels were calculated using the 2^−ΔΔCt^ method, with TATA-binding protein (TBP) as the internal control with 3–4 biological replicates per group. Primer sequences are provided in (**Fig. S2**).

### 2.10 Western blot

Whole-cell lysates were collected from iPSC-CM in different groups, Glu, Gal, and Gal + TCA in 1× PBS, and proteins were extracted using RIPA buffer (ThermoFisher). Protein concentration was determined using Bradford assay. Equal amounts of protein (40 µg per sample) were separated by SDS–PAGE and transferred to nitrocellulose membranes. Membranes were blocked in skim milk for 1 h at room temperature and then incubated overnight at 4°C with primary antibodies against PLN (Cell Signaling Technology), SERCA2a (Santa Cruz Biotechnology), or GAPDH (Proteintech). After three washes with TBS-T (10 min each), membranes were incubated with HRP-conjugated secondary antibodies (1:3000) for 1 h at room temperature. Bands were visualized using Clarity™ Western ECL substrate (Bio-Rad) and imaged on a ChemiDoc system (Bio-Rad). Band intensities were quantified using Image Lab software (Bio-Rad, v6.0.1).

### 2.11 Wheat germ agglutinin (WGA) membrane staining

iPSC-CM in different groups, Glu, Gal, and Gal + TCA were fixed in 4 % formaldehyde in PBS for 10 min and then washed with PBS (3 × 3 min). After blocking with 2 % BSA in PBS for 20 min, cells were incubated with 5 nM WGA Alexa Fluor 488 conjugate (ThermoFisher) diluted in 2 % BSA/TBS for 30 min at 37°C. Cells were then washed with PBS (2 × 3 min) and nuclei were counterstained with Hoechst 33,342 (0.5 µg/mL, ThermoFisher) for 5 min, followed by two additional washes with PBS. Images were acquired using a Cytation 10 multimodal imaging system (BioTek), and cell area was quantified from the acquired images using BioTek Gen5 software.

### 2.12 Cellular bioenergetics assessment (OCR/ECAR)

*a) Mito stress test*

Oxygen consumption rate (OCR) was measured using Seahorse XFe24 Analyzer (Agilent Technologies). The iPSC-CM were seeded at 100,000 cells/cm² in XF microplates and cultured for 7 days in different groups, Glu, Gal, and Gal + TCA. The media were replaced with XF base DMEM supplemented with glucose, GlutaMAX™, and pyruvate, followed by a 1-hour incubation at 37 °C. The OCR was recorded under basal conditions and after sequential injection of oligomycin (1 µM), CCCP (2 µM), and a mix of rotenone and antimycin A (1 µM each). The maximal respiration rate was calculated as OCR post-CCCP minus non-mitochondrial OCR. The spare respiratory capacity was the difference between maximal and basal respiration, and ATP-linked respiration was defined as basal OCR minus OCR post-oligomycin. Each condition included 5–6 biological replicates. Data were normalized to total protein content and expressed as pmol OCR/min/µg protein.

*b) Glycolysis stress test*

Extracellular acidification rate (ECAR) was measured using the Seahorse XFe24 Analyzer. iPSC-CM were cultured for 7 days in Glu, Gal, and Gal + TCA groups, then transferred to XF base DMEM (glucose-free, pH 7.4) with 1X GlutaMAX™ for 1 hour at 37 °C without CO₂. The ECAR was recorded at baseline (non-mitochondrial respiration) and following sequential injections of 10 mM glucose, 1 μM oligomycin, and 50 mM 2-deoxy-D-glucose (2-DG). Glycolysis was calculated as ECAR post-glucose addition minus baseline, glycolytic capacity as ECAR post-oligomycin addition minus baseline, and glycolytic reserve as the difference between post oligomycin and glucose addition. Each group included 6–7 biological replicates, and results were normalized to protein content and expressed as ECAR picomoles/min/μg protein.

### 2.13 Statistics

Statistical analyses were carried out using R Studio (v4.2.0) and GraphPad Prism (v9.02). We performed an unpaired two-tailed Student’s *t*-test for comparisons between two groups. For experiments with three groups (Glu, Gal, and Gal + TCA), one-way ANOVA followed by Tukey’s multiple-comparisons test was applied. For MMP and LDH dose-screening experiments, one-way ANOVA followed by Dunnett’s post hoc test was used to compare each TCA metabolite concentration with the control. A p-value < 0.05 was considered statistically significant. All experiments included at least three independent biological replicates.

## 3 Results

### 3.1 Targeted metabolomic analysis reveals glycolytic bias and limited TCA cycle activity in iPSC-CM

**Fig. 1:**
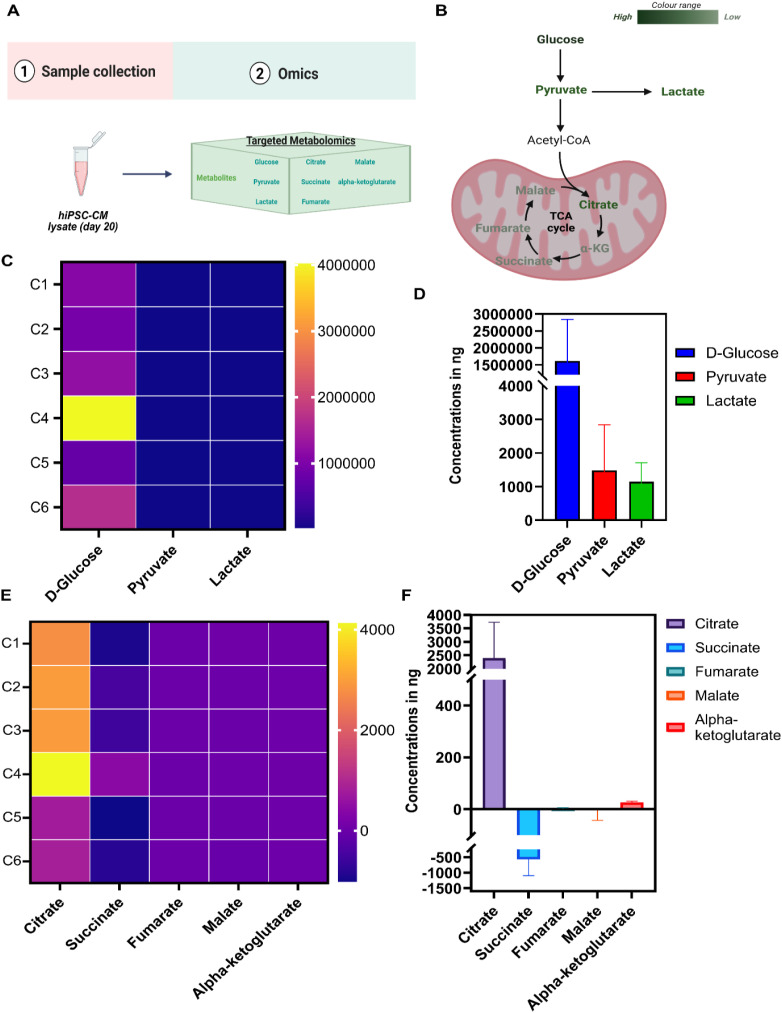
**. Targeted metabolomic profiling of iPSC-CM reveals changes in glycolysis and TCA cycle activity.** A) Schematic overview of the experimental workflow. iPSC-CM pellets were collected for metabolite extraction and targeted metabolomic analysis (n = 6). B) Flowchart showing relative abundance of glycolytic and TCA cycle metabolites. Dark green indicates high abundance, light green indicates low abundance, and black denotes undetected metabolites. C) Heatmap of glucose, pyruvate, and lactate levels across biological replicates, with color intensity reflecting metabolite abundance. D) Histograms showing quantification of glucose, pyruvate, and lactate levels. E) Heatmap of TCA cycle intermediates including citrate, succinate, fumarate, malate, and α-ketoglutarate. Abundance is visualized using a color scale from yellow/orange (high) to violet/blue (low). F) Histograms showing quantification of TCA cycle intermediates. Data is mean±SD. C(1–6) denotes individual biological replicates of iPSC-CMs.

The human iPSC were differentiated into iPSC-CM and characterized for pluripotency and cardiac lineage respectively (**Fig. S3A–B**). The iPSC-CM were subjected to targeted metabolomic analysis with a focus on glycolytic and TCA cycle intermediates (Fig. 1A). The quantitative analysis of day 21 iPSC-CM revealed elevated glucose levels, accompanied by downregulation of pyruvate and lactate, indicating a glycolysis-dominant metabolic state (**Fig. 1B–D**), which is a typical signature of fetal cardiomyocytes. Furthermore, intracellular profiling of TCA cycle intermediates revealed that citrate was the most abundant metabolite detected in iPSC-CM, while other intermediates of TCA cycle including succinate, malate, fumarate, and α-ketoglutarate were present but at very low concentrations (**Fig. 1E, F**). These data indicate limited engagement of TCA cycle in iPSC-CM and confirm that these cells have an immature phenotype with metabolic activity similar to that of fetal cardiomyocytes.

### 3.2 Dose optimization for TCA cycle intermediates and evaluation of their effect on electrophysiological maturation of iPSC-CM

**Fig. 2:**
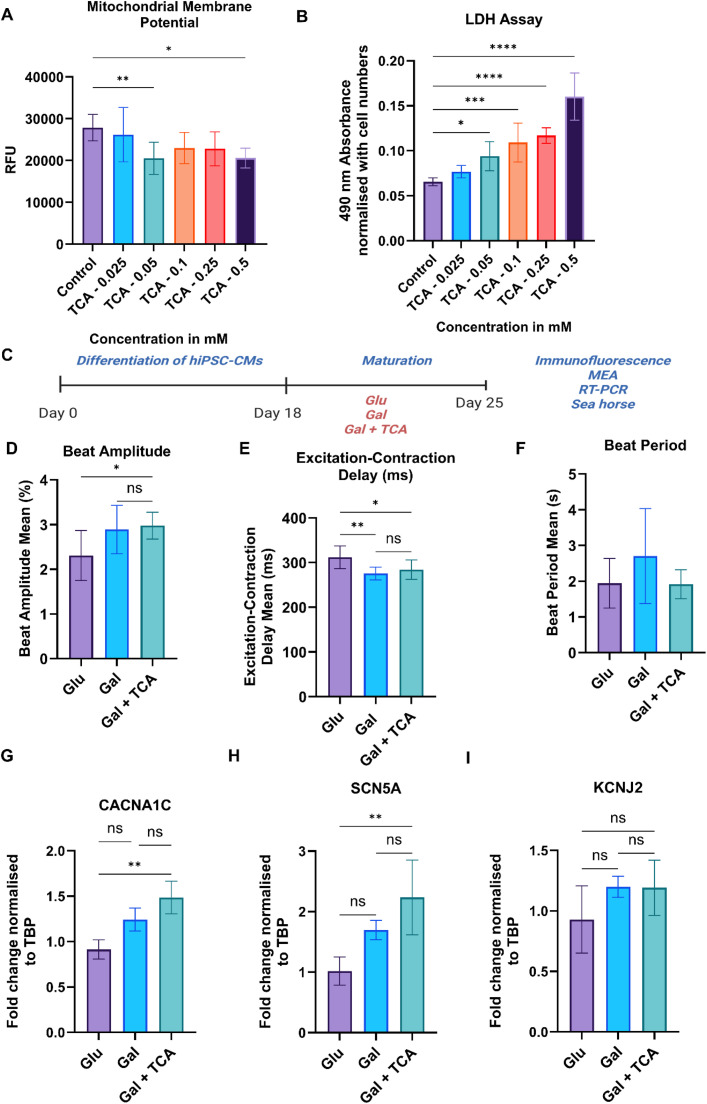
**. Effect of TCA cycle metabolites in electrophysiological properties of iPSC-CM.** A) Dose optimization of TCA cycle intermediates (succinate, fumarate, malate, and α-ketoglutarate), the iPSC-CM were treated with TCA cycle metabolites at different doses (0.025 mM, 0.05 mM, 0.1 mM, 0.25 mM and 0.5 mM) and MMP was measured using TMRE assay. Relative fluorescence units (RFU) reflect mitochondrial polarization (n = 5–6 per group). B) Cell viability assessed by LDH assay in iPSC-CM after treatment with TCA cycle metabolites at different concentrations (n = 5–6 per group). C) Schematic overview of the experimental design. iPSC-CM were cultured for 7 days in glucose-rich media (Glu), galactose-rich media (Gal), and galactose rich media supplemented with TCA cycle intermediates (Gal + TCA). D–F) MEA analysis of iPSC-CM in different groups to measure, beat amplitude (D), excitation-contraction delay (E), and beat period (F) (n = 6–8). G-I) q-PCR analysis of genes SCN5A, CACNA1C, and KCNJ2 which encode for key ion channels NaV1.5, CaV1.2 and Kir2.1 respectively, in iPSC-CM cultured for 7 days in Glu, Gal, and Gal + TCA media (*n* = 3–4 for each group). Relative expression levels were normalized to the housekeeping gene (TBP) and compared across groups. Data are presented as mean ± SD. *p < 0.05, **p < 0.002, ***p < 0.0002, ****p < 0.0001).

As mentioned above our targeted metabolomics suggest that there was low abundance of TCA cycle intermediates in iPSC-CM, therefore we hypothesized that supplementation of these metabolites (succinate, fumarate, malate, and α-ketoglutarate) to iPSC-CM would switch them toward a mature phenotype. However, to find optimal doses of these supplements we performed a dose response curve and studied the effect of different concentrations (0.025 mM, 0.05 mM, 0.1 mM, 0.25 mM and 0.5 mM) of these intermediates on iPSC-CM. We chose this micromolar range for optimization, because reported physiological concentrations of circulating TCA intermediates are typically in the low-to-mid µM range. The end points chosen were mitochondrial membrane potential (MMP) for mitochondrial health and lactate dehydrogenase assay for cytotoxicity of these metabolites toward iPSC-CM. Based on this analysis the dose of TCA cycle intermediates chosen for further experiments was 0.025 mM. At this dose we did not find any significant change in MMP compared to control group, therefore, treatment at this dose preserved mitochondrial integrity of iPSC-CM (Fig. 2A), also this dose did not cause any cytotoxicity in iPSC-CM (**Fig. 2B**).

To identify an appropriate control (or basal) condition, we compared two media formulations commonly used in the literature with exogenous fatty acid supplementation: Glu-rich (10 mM glucose) and physiologic glucose concentration (Phy Glu) (5.5 mM glucose) [27]. After 7 days of culture, qPCR showed no significant differences in genes related to metabolism (PGC1A, PPARA, AMPK) or electrophysiology (SCN5A, ATP2A2, KCNJ2) between the two conditions (**Fig. S4**). Therefore, we used Glu-rich (10 mM glucose + fatty acids) media as the control for all subsequent experiments to ensure consistency in all the assays. Also, we chose a 7-day treatment protocol because this exposure time is sufficient to elicit transcriptional and functional remodeling [28,29].

To assess the effects of TCA cycle metabolites supplementation on cardiomyocyte maturation, iPSC-CM were cultured for seven days in glucose rich media (Control), galactose rich media (Media Control), and galactose rich media supplemented with TCA cycle intermediates (succinate, fumarate, malate, and α-ketoglutarate) at a dose of 0.025 mM as detailed in the method (**Fig. 2C**). The electrophysiological measurements were taken using multi-electrode array. The iPSC-CM treated with galactose + TCA cycle metabolites exhibited enhanced electrophysiological properties. We observed a significant increase in beat amplitude (**Fig. 2D**) and a decrease in excitation–contraction delay (**Fig. 2E**) in iPSC-CM treated with TCA cycle supplements compared to the control group. We did not observe any change in beat period among different groups (**Fig. 2F**). In MEA recordings, spike amplitude reflects the strength and synchrony of electrical activity in the iPSC-CM monolayer, whereas excitation–contraction delay measures the time between electrical activation and the onset of contraction. Thus, the increased amplitude and reduced delay observed with TCA supplementation are consistent with improved electrical propagation and electromechanical coupling which are features of mature iPSC-CM, while the unchanged beat period indicates that the spontaneous beating rate was not altered. These results demonstrate that treatment of iPSC-CM with TCA cycle metabolites improved their electrophysiological properties and maturation.

To explore the mechanism underlying the improved electrophysiological function, we performed qPCR to assess the expression of genes SCN5A, CACNA1C, and KCNJ2 which encode for key ion channels NaV1.5, CaV1.2 and Kir2.1 respectively (**Fig. 2G-I**). The expression of SCN5A and CACNA1C was higher in the Gal + TCA group compared to the control group, whereas KCNJ2 gene expression was unaffected. Upregulation of ion channel genes in Gal + TCA group resulted in a shorter excitation–contraction delay and increased spike amplitude compared to Glu group, promoting iPSC-CM maturation [30,31].

### 3.3 Effect of TCA cycle intermediate supplementation on calcium handling and morphology of iPSC-CM

**Fig. 3:**
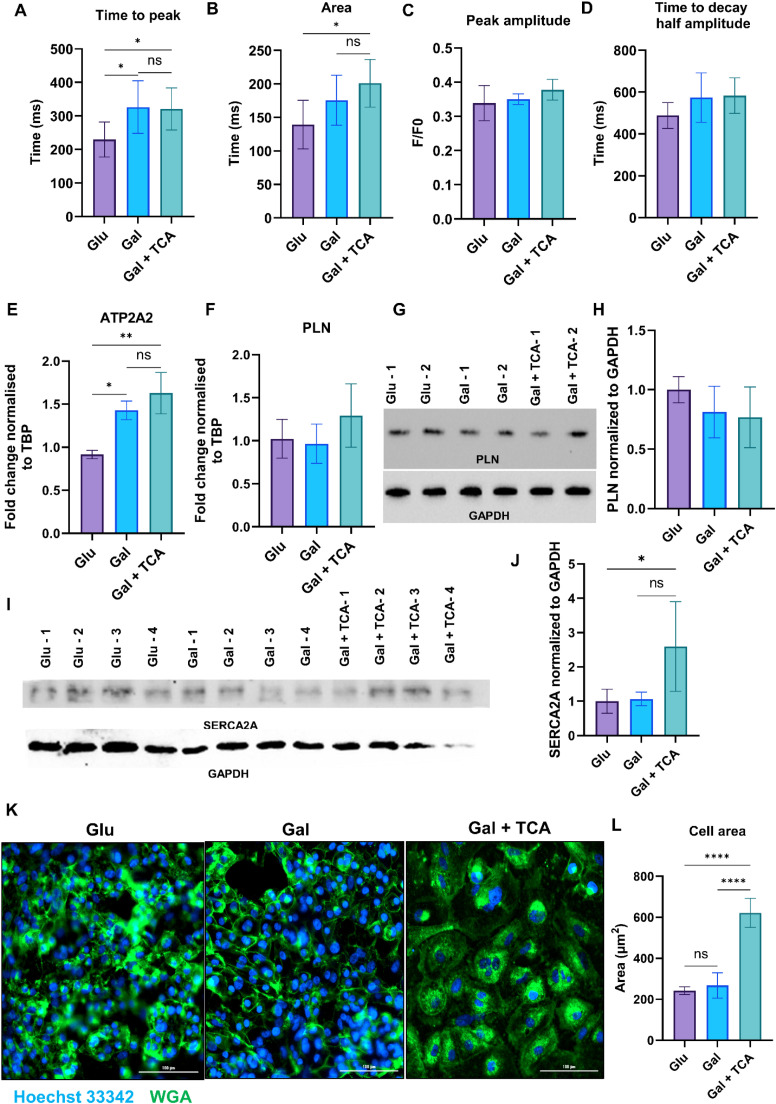
**. Effect of TCA cycle metabolites supplementation on calcium handling and morphology of iPSC-CM.** A, B) Quantitative analysis of calcium transient kinetics in iPSC-CM after 7 days in culture in glucose rich media (Glu), galactose rich media (Gal), and galactose rich media supplemented with TCA cycle intermediates (succinate, fumarate, malate, and α-ketoglutarate) at a concentration of 0.025 mM (Gal+TCA). There was an improvement in the (A) Time to peak, (B) Area under the curve and no change in the (C) Peak amplitude and (D) Time to decay half amplitude after supplementation of TCA cycle intermediates, reflecting an improvement in the total calcium transient activity. (n = 6–7). E, F) q-PCR analysis of genes associated with calcium handling (ATP2A2, and PLN) in iPSC-CM cultured for 7 days in different media compositions (*n* = 3–4 for each group). Relative expression levels were normalized to the housekeeping gene (TBP) and compared across groups. G-J) Representative Western blots of iPSC-CM in different groups, the membranes were probed for PLN and SERCA2A antibodies. GAPDH was used as a loading control. Densitometry quantification of protein levels was normalized to GAPDH. K) Representative immunofluorescence images of WGA (green) and Hoechst 33,342 staining (blue, nucleus) showing cellular area in iPSC-CM in different groups. Images were acquired at 40× magnification. L) Quantification of cell area in different groups (n=5, 75 to 170 cells per group). Values are presented as mean ± SD. Statistical significance was determined by one-way ANOVA with Tukey’s multiple comparison test (p < 0.05).

Calcium plays an important role in the contraction of adult cardiomyocytes, and calcium handling in cardiomyocytes is very important for the functioning of mature and adult cardiomyocytes. To assess the effects of TCA cycle intermediates supplementation on calcium handling, calcium transient kinetics was conducted in iPSC-CM cultured for seven days in glucose rich media, galactose rich media, and galactose rich media + TCA intermediates (succinate, fumarate, malate, and α-ketoglutarate) at a concentration of 0.025 mM. In the Gal + TCA group, calcium transients showed a longer time to peak and a greater area under the curve compared to controls (Fig. 3A–B), while peak amplitude and half-decay time were unchanged in all the groups (**Fig. 3C–D**). Overall, these results suggest that TCA cycle intermediates supplementation increase intracellular Ca²⁺ availability during contraction which is an indicator of a shift toward adult phenotype with more mature calcium handling. To investigate the mechanism, we assessed calcium-handling regulators in iPSC-CM cultured in Glu, Gal, and Gal + TCA. Our qPCR data showed that ATP2A2 (SERCA2a) expression increased in both Gal and Gal + TCA compared with Glu group, whereas PLN gene expression was unchanged in all the groups (**Fig. 3E–F**). Similarly, western blot analysis showed that SERCA2a expression increased in Gal + TCA compared to Glu group, whereas PLN expression was unchanged across all the groups (**Fig. 3G–J**). In the current study, ATP2A2 (SERCA2a) upregulation suggests an increased capacity for sarcoplasmic reticulum (SR) Ca²⁺ reuptake, which supports both relaxation and SR refilling for subsequent contractions. Because PLN is a key inhibitory regulator of SERCA2a (with inhibition relieved mainly by PLN phosphorylation), unchanged PLN transcript levels alongside higher ATP2A2 is an indicator of a shift toward greater SR Ca²⁺ cycling potential, which may lead to a greater Ca²⁺ availability during contraction even if decay kinetics are not measurably faster at this stage [32,33].

To determine whether these improvements in calcium handling were accompanied by morphological changes in iPSC-CM, we performed wheat germ agglutinin staining (WGA) to determine the area of iPSC-CM. The iPSC-CM supplemented with TCA cycle intermediates displayed a significant increase in cell area compared to the cells in both Glu and Gal control groups (**Fig. 3K,L**). Together, these findings indicate that TCA cycle metabolites supplementation improves calcium handling efficiency and cellular morphology of iPSC-CM towards a more mature phenotype.

### 3.4 Effect of TCA cycle intermediates supplementation on mitochondrial bioenergetics and glycolytic reserve in iPSC-CM

**Fig. 4:**
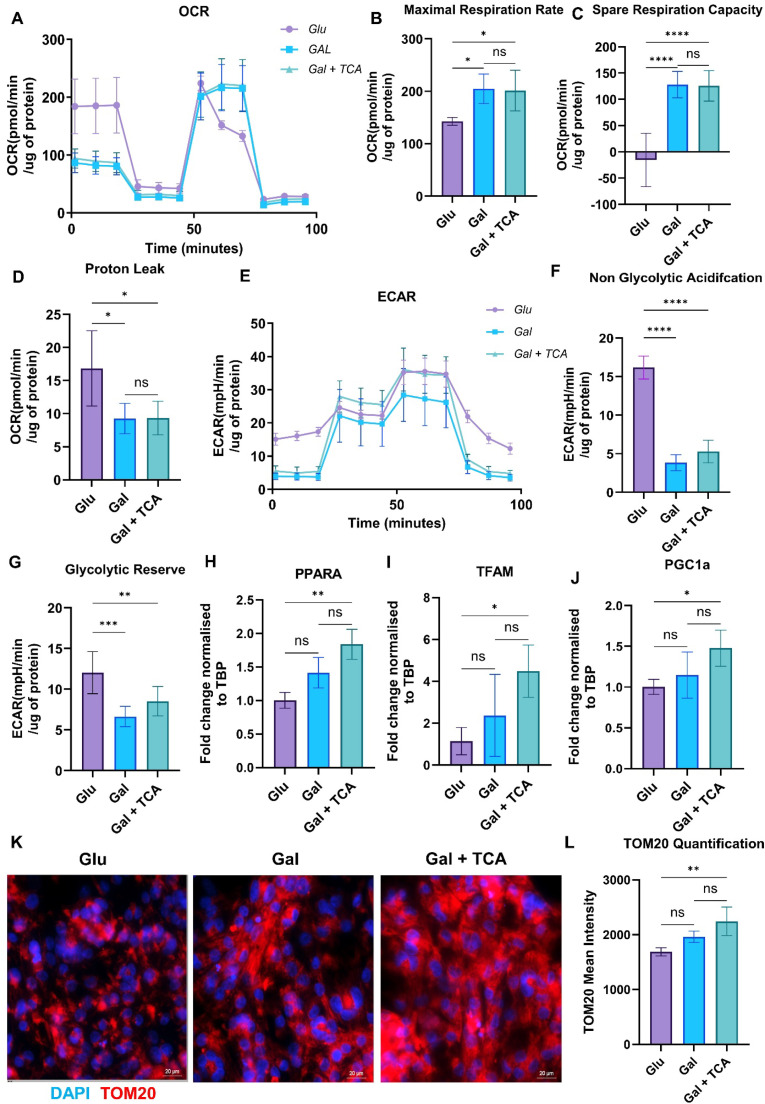
**Effect of TCA cycle metabolites supplementation on mitochondrial bioenergetics of iPSC-CM.** A) Real-time oxygen consumption rate (OCR) in iPSC-CM after 7 days in culture in glucose rich media (Glu), galactose rich media (Gal), and galactose rich media supplemented with TCA cycle intermediates (Gal + TCA) (n = 5,6). The OCR was measured over time using a mitochondrial stress test protocol and all values were normalized to total protein content to account for differences in cell density. The following key mitochondrial bioenergetics parameters were derived: B) Maximal Respiration Rate, C) Spare Respiratory Capacity, D) Proton Leak from OCR profiles. E) Extra Cellular Acidification Rate (ECAR) in iPSC-CM, F, G) Glycolytic Reserve and Non-Glycolytic Acidification were calculated from ECAR profiles. H-J) q-PCR analysis of genes associated with mitochondrial biogenesis (PPARA, PGC1a and TFAM) was performed in iPSC-CM cultured for 7 days in different groups (*n* = 3–4 for each group).K) Representative immunofluorescence images of TOM20 (red) and DAPI staining (blue, nucleus) showing mitochondrial abundance in iPSC-CM after 7 days in culture in different groups. I) Quantification of mean intensity of TOM20 staining in iPSC-CM in different groups. Images were acquired at 60× magnification. Data are expressed as mean ± SD. Statistical analysis was performed using one-way ANOVA followed by Tukey’s multiple comparisons test (*-p < 0.05, **-p < 0.002, ***p < 0.0002, ****-p < 0.0001).

Mitochondria provide energy for cell growth, development of specialized functions and contraction of cardiomyocytes. Therefore, mitochondrial bioenergetics play an important role in the maturation of iPSC derived cardiomyocytes. Hence, to further investigate the effect of TCA cycle intermediates supplementation on maturation of iPSC-CM, we performed Seahorse analysis to assess mitochondrial bioenergetics. The oxygen consumption rate (OCR) was measured in iPSC-CM cultured in glucose rich media, galactose rich media, and galactose rich media + TCA intermediates (succinate, fumarate, malate, and α-ketoglutarate) at a concentration of 0.025 mM. The OCR values were normalized to total protein to account for variability in cell number (Fig. 4A). The treatment with TCA cycle intermediates led to a significant increase in maximal respiration compared to control groups, indicating a shift toward enhanced mitochondrial oxidative phosphorylation for energy production in cells (Fig. 4B). Also, we observed a significant increase in spare respiratory capacity and a decrease in proton leak in iPSC-CM supplemented with TCA cycle metabolites, which reflects a greater metabolic flexibility under conditions of high energy demand during the process of iPSC-CM maturation (Fig. 4C**,**D). These findings demonstrate that TCA cycle intermediates supplementation improves mitochondrial function and supports a shift toward a mature phenotype of iPSC-CM that relies on oxidative phosphorylation for ATP production and energy demands.

The fetal cardiomyocytes and immature iPSC-CM rely on glycolysis for energy demands and functioning of the cells. As described above after treatment with TCA cycle intermediates there was a shift in metabolic pathways towards oxidative phosphorylation. Therefore, we wanted to study the effect of supplementation of TCA cycle metabolites on glycolytic capacity and reserve. The extracellular acidification rate (ECAR) was measured in iPSC-CM treated with glucose rich media, galactose rich media, and galactose rich media + TCA intermediates (succinate, fumarate, malate, and α-ketoglutarate) at a concentration of 0.025 mM. The ECAR values were normalized to total protein content to account for cell density and used to calculate the glycolytic reserve, and non-glycolytic acidification (Fig. 4E). Our data demonstrate that there was a significant decrease in glycolytic reserve and non-glycolytic acidification rate in TCA cycle intermediates supplemented iPSC-CM (Fig. 4F**,**G). This suggests that glycolysis was not the preferred metabolic process to meet the energy demands, which is evidence for the metabolic maturation of iPSC-CM after treatment with TCA cycle metabolites.

To determine whether improved metabolism was accompanied by changes in mitochondrial biogenesis and content, we performed qPCR for PPARA, PGC1A, and TFAM in iPSC-CM cultured in Glu, Gal, and Gal + TCA (Fig. 4H**–**J). The gene expression of PPARA, PGC1A (PGC-1α), and TFAM were significantly upregulated in Gal + TCA compared with Glu. The PPARA/PGC-1α regulate transcriptional programs supporting oxidative metabolism and mitochondrial biogenesis [34], while TFAM is essential for mtDNA maintenance, transcription, and replication [35]. In parallel, we assessed mitochondrial abundance by TOM20 immunofluorescence and quantitative image analysis. TOM20 is a marker for outer mitochondrial membrane and was used as a proxy for mitochondrial content to compare relative mitochondrial abundance in different groups. The mean TOM20 intensity was higher in iPSC-CM cultured in Gal + TCA compared with Glu (Fig. 4K). Collectively, these findings indicate that TCA cycle supplementation promotes a metabolic shift from glycolysis to oxidative phosphorylation for mitochondrial respiration and to meet the energy demands of mature iPSC-CM for contraction and functioning.

### 3.5 Effect of TCA cycle intermediate supplementation on sarcomere length, metabolic and structural gene expression in iPSC-CM

**Fig. 5:**
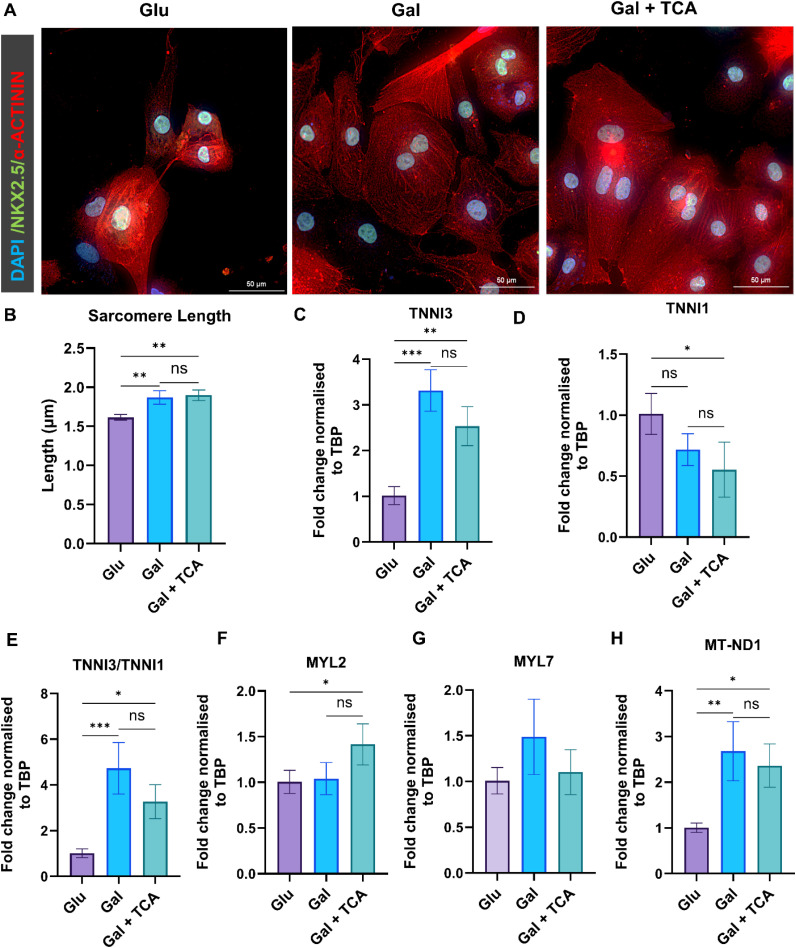
**Impact of TCA cycle metabolites supplementation in sarcomere length and expression of iPSC-CM maturation genes.** A) Representative immunofluorescence images showing α-actinin (red) and nuclei stained with Hoechst 33,342 (blue) showing sarcomere in iPSC-CM after 7 days in culture in glucose rich media (Glu), galactose rich media (Gal), and galactose rich media supplemented with TCA cycle intermediates (Gal + TCA). B) Sarcomere length was quantified by measuring 3–6 sarcomere units per cell from 5–10 cells per image, yielding 30 sarcomere measurements per group, which were averaged to report mean sarcomere length (A, n = 3). Images were acquired at 60× magnification. C–H) q-PCR analysis of genes associated with sarcomeric structure (TNNI3, TNNI1, TNNI3/TNNI1 ratio, MYL2, MYL7) and cellular metabolism (MT-ND1) in iPSC-CM cultured for 7 days in Glu, Gal, and Gal + TCA media (*n* = 3–4 for each group). Relative expression levels were normalized to the housekeeping gene (TBP) and compared across groups. Data are presented as mean ± SD. Statistical significance was assessed using one-way ANOVA followed by Tukey’s multiple comparisons test (*-p < 0.05, **-p < 0.002 & ***p < 0.0002).

Structural maturation of iPSC-CM is commonly assessed by sarcomere length (Z-line spacing), which increases and becomes more uniformly organized as myofibrils assemble and align toward an adult-like contractile architecture [36]. Therefore, we assessed sarcomeric length using Nikon NIS elements software after 7 days under each culture condition using α-actinin immunostaining (Fig. 5A, B). Our results indicated that sarcomere length increased in iPSC-CM maintained in Gal and Gal + TCA compared with Glu (Fig. 5B) indicating improved structural maturation of iPSC-CM. To further confirm that treatment with TCA cycle intermediates improved metabolic and structural maturation of iPSC-CM, we analysed the expression of several downstream genes involved in these pathways. qPCR was performed on RNA samples from iPSC-CM cultured for seven days in glucose rich media, galactose rich media, and galactose rich media + TCA intermediates (succinate, fumarate, malate, and α-ketoglutarate) at a concentration of 0.025 mM. The expression of adult troponin isoform TNNI3 was significantly upregulated and TNNI1 expression was downregulated and the ratio of TNNI3/TNNI1 is significantly upregulated in Gal + TCA treated iPSC-CM compared to Glu (Fig. 5C**-**E). The TNNI3 is an isoform that is more predominantly present in adult cardiomyocytes, and TNNI1 is present in fetal cells [37], therefore these results strongly suggest a switch from fetal toward a more mature and adult phenotype of iPSC-CM in the cells treated with TCA intermediates. Furthermore, the expression of MYL2 gene was increased in TCA cycle intermediates treated iPSC-CM, whereas MYL7 expression remained unchanged across all the groups (Fig. 5F,G). The MYL2 gene is highly abundant in adult cardiomyocytes and MYL7 is present in fetal cells [38]. We also found a significant increase in MT-ND1 expression in iPSC-CM treated with TCA cycle metabolites (Fig. 5H). The MT-ND1 encodes a mitochondrial DNA–encoded subunit of Complex I, a core component of the oxidative phosphorylation (OXPHOS) system [39]. Therefore, these findings suggest that TCA cycle metabolite supplementation promotes metabolic and structural maturation of iPSC-CM.

### 3.6 Validation of TCA metabolite uptake, comparative effects of individual intermediates in iPSC-CM and mechanisms

**Fig. 6:**
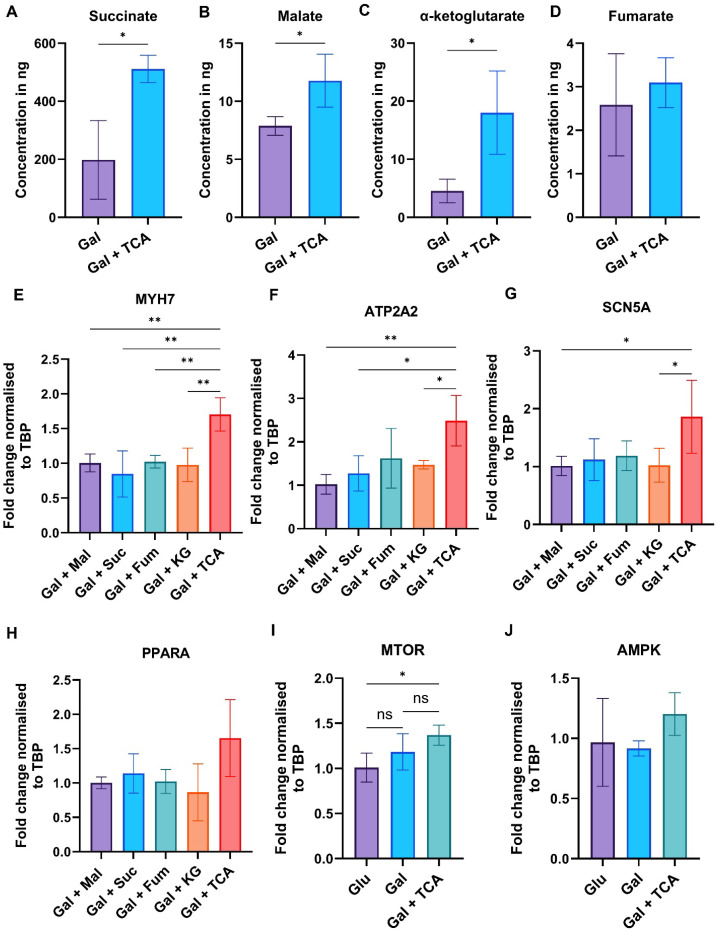
**Metabolites uptake and effects of individual and combined TCA intermediates on the maturation of iPSC-CM.** (A–D) Targeted metabolomics was performed in whole-cell lysates of iPSC-CM cultured for 7 days in Gal or Gal + TCA (n = 3). Intracellular concentrations of succinate, malate, α-ketoglutarate, and fumarate were normalised to cell number and reported in nanograms (ng). The concentration of these metabolites was significantly higher in Gal + TCA group compared with Gal alone group, confirming the uptake of the supplemented metabolites. (E–H) qPCR analysis was performed to compare the effects of combination and individual metabolites supplementation on maturation of iPSC-CM. Expression of maturation-associated markers spanning ion channel, calcium-handling, structural, and mitochondrial signaling (SCN5A, ATP2A2, MYH7, and MYL2) was assessed (n = 3–4). (I–J) qPCR analysis of AMPK and mTOR was assessed to understand potential signaling mechanisms associated with metabolic maturation of iPSC-CM (n=3–4). Relative expression levels were normalized to the housekeeping gene (TBP) and compared across groups. Data are presented as mean ± SD. Statistical significance was assessed using one-way ANOVA followed by Tukey’s multiple comparisons test (*-p < 0.05 & **-p < 0.002).

To determine whether the supplemented TCA cycle intermediates were taken up by iPSC-CM, we performed targeted metabolomics after 7 days of culture in Gal or Gal + TCA conditions. Intracellular concentrations of succinate, malate, α-ketoglutarate, and fumarate were quantified. Our data demonstrate that the levels of these metabolites were significantly higher in Gal + TCA group compared to Gal only group, demonstrating effective uptake of the supplemented metabolites (Fig. 6A–D). Next, to determine whether the effects were driven by a single metabolite or by the combined cocktail, we investigated the effects of each metabolite individually in different groups by qPCR for maturation-associated markers (MYH7, ATP2A2, SCN5A and PPARA) (Fig. 6E–H). Our data demonstrate that the expression of different maturation associated markers, MYH7, ATP2A2 and SCN5A was significantly higher when TCA cycle metabolites (succinate, fumarate, malate, and α-ketoglutarate) were supplemented together compared to individual metabolites supplementation. These data show that supplementation of combination of these four metabolites is required for maturation of iPSC-CM. Furthermore, TCA cycle intermediates influence several cell signaling pathways related to development and phenotype of the cells. In this regard AMPK and mTOR are linked to metabolic remodeling of the cells by integrating cellular nutrients, therefore, we evaluated the expression of both AMPK and mTOR after supplementation of TCA cycle intermediates (Fig. 6I–J). The mTOR expression was significantly higher in Gal + TCA supplemented cells compared with Glu, whereas AMPK levels were unchanged. Together, these data suggest that in addition to promoting oxidative metabolism, TCA cycle intermediates supplementation engages mTOR-associated metabolic remodeling to promote mature phenotype of iPSC-CM.

## 4 Discussion

iPSC derived cardiomyocytes offer great promise for disease modeling, drug discovery and as a source of cell therapy. However, their immature phenotype is a major hurdle in clinical application of iPSC-CM based therapies [40]. This study demonstrates that supplementation with TCA cycle intermediates enhances the metabolic, structural, and functional maturation of iPSC-CM. Our targeted metabolomic analysis of iPSC-CM revealed a downregulation in the levels of four TCA cycle intermediates, succinate, fumarate, malate, and α-ketoglutarate. The supplementation of these metabolites at a dose of 0.025 mM promoted structural and functional maturation of iPSC-CM. This dose was selected based on our optimization experiments, additionally the reported physiological concentrations of circulating TCA cycle intermediates are typically in the low-to-mid µM range [41]. The electrophysiological remodeling is one of the key processes in the functional maturation of cardiomyocytes [42]. Our data suggest that treatment with TCA cycle supplements exhibited reduced excitation-contraction delay, increased beat amplitude, and stabilized beat period in iPSC-CM compared to the cells cultured in Glu. These findings indicate improvement in electrical conduction and contractile function, which are hallmarks of mature and adult phenotype of cardiomyocytes. Furthermore, treatment with TCA cycle intermediates improved the calcium handling properties of iPSC-CM as we observed prolonged time to peak and increased area under the curve compared to control group. These changes point to a slower but more sustained calcium influx in iPSC-CM, potentially reflecting enhanced sarcoplasmic reticulum calcium loading or modulation of L-type calcium channel activity in adult cardiomyocytes as evidenced in our PCR and western analysis [43,44].

Mitochondria play a very important role in the maturation of cells. In cardiomyocytes during development from fetal to adult phenotype, there is a shift from glycolytic metabolism in early development to an oxidative metabolism in adult and mature cells. This transition in energy metabolism involves structural changes in the mitochondria. Furthermore, mitochondria are the powerhouse of the cells and provide energy for growth and contraction of cardiomyocytes. Therefore, mitochondrial bioenergetics play crucial role for maturation of iPSC derived cardiomyocytes [45]. In the current study, we observed that supplementation with TCA cycle intermediates increased maximal respiration and spare respiratory capacity, which is an indicator of improved oxidative phosphorylation and energy responsiveness. Interestingly, we observed a significant decrease in glycolytic reserve and non-glycolytic acidification in TCA cycle supplements treated iPSC-CM that further support a shift from glycolysis toward oxidative metabolism. Furthermore, this shift in energy metabolism was associated with an upregulation in the expression of cardiomyocyte maturation genes TNNI3, MYL2 and downregulation in the expression of fetal gene TNNI1. Also, we observed an increase in MT-ND1 levels, indicating that TCA cycle metabolite supplementation promotes metabolic and structural maturation of iPSC-CM.

Furthermore, our data also demonstrate that the effect of combination of above listed four TCA cycle intermediates was superior to individual metabolites. In addition to serving as substrates that support mitochondrial oxidative metabolism, these TCA cycle intermediates are also known to activate several cell signaling pathways that influence cardiomyocyte development and phenotype. Our data demonstrate that supplementation of TCA cycle intermediates was associated with an increase in mTOR expression, interestingly mTOR is reported to enhance metabolic remodeling of cells. Furthermore, succinate and fumarate have been shown to stabilize hypoxia-inducible factor-1α (HIF-1α) through inhibition of prolyl hydroxylase enzymes that further affect mitochondrial metabolism in the cells [46]. Succinate is also reported to act as an extracellular signaling molecule by engaging the succinate receptor SUCNR1 (GPR91), triggering downstream signaling pathways independent of its metabolic role [47]. Furthermore, α-ketoglutarate serves as an essential co-substrate for α-KG-dependent dioxygenases, including TET DNA demethylases and JmjC-domain histone demethylases, thereby influencing epigenetic regulation and gene expression programs associated with cellular maturation [48]. Also, fumarate has been reported to activate NRF2-dependent antioxidant signaling through the modification of KEAP1, linking TCA cycle metabolism to redox-sensitive transcriptional responses [49]. Therefore, the supplementation of TCA cycle metabolites in the current study may have been improving the iPSC-CM maturation by enhancing metabolite-driven cell signaling. However, further studies are warranted to explore the depth of cell signaling mechanisms that promote TCA cycle metabolites mediated maturation of iPSC-CM. Even though in the current study, Gal + TCA supplementation promoted maturation of iPSC-CM, these improvements were generally modest compared to only Gal group and in many experiments, it did not reach statistical significance. This is likely because galactose supplementation alone may have driven iPSC-CM toward oxidative metabolism, leaving a smaller incremental window for additional benefit with TCA cycle supplements. Future studies should systematically optimize the dose, timing, and combination of different intermediates to better understand the beneficial effects of supplementation of TCA cycle intermediates. In addition, incorporating higher-resolution maturation readouts such as patch-clamp mediated action potential measurements, electron microscopy, and detailed Ca²⁺ reuptake kinetics may detect further improvements that can complement the outcome of assays performed in the current study and add further depth to the mechanism. Finally, although metabolite supplementation improved metabolic and functional maturation compared to control groups, this study did not directly quantify how closely these cells mimic fully mature adult human ventricular cardiomyocytes. Future studies should focus on direct comparison of functional maturation of iPSC-CM after these interventions with adult human cardiomyocytes. Regardless, this study provides avenues to plan strategies for physiologically relevant comprehensive and scalable approaches to promote metabolic and functional maturation of iPSC-CM through TCA cycle intermediate supplementation.

## Author contributions

The study was conceptualized and designed by KNA and SD. KNA, EB, MG carried out the experiments and acquired the data. KNA and SD interpreted the data and performed statistical analysis. MA carried out metabolomics analysis, interpreted the data and performed statistical analysis. KNA and SD drafted the manuscript. All authors have read and approved the final manuscript.

## Competing interests

All authors declare that there are no financial relationships with any organizations that might have an interest in the submitted work. Authors declare no other relationships or activities that could appear to have influenced the submitted work.

**Data availability**

N

N

All data needed to evaluate the conclusions in the paper are present in the paper and supplementary materials.

**Data sharing**

The data that supports the findings of this study will be available from the corresponding author upon reasonable request.

## CRediT authorship contribution statement

**Keshav Narayan Alagarsamy:** Writing – review & editing, Writing – original draft, Methodology, Investigation, Formal analysis, Data curation, Conceptualization. **Emilee Bueckert:** Writing – original draft, Methodology, Investigation, Formal analysis, Data curation. **Mehak Gupta:** Methodology, Investigation. **Michel Aliani:** Methodology, Investigation. **Sanjiv Dhingra:** Writing – review & editing, Validation, Supervision, Resources, Project administration, Funding acquisition, Formal analysis, Conceptualization.

## Declaration of competing interest

The authors declare that they have no known competing financial interests or personal relationships that could have appeared to influence the work reported in this paper.

## Data Availability

Data will be made available on request.

## Appendix Supplementary materials

Image, application 1
