# Supplementary material for: TCA cycle intermediates as an adjunct strategy for human iPSC-derived cardiomyocyte maturation
Source: BBA Adv. 2026 Feb 16;9:100183. doi: 10.1016/j.bbadva.2026.100183 (PMC12930173; doi:10.1016/j.bbadva.2026.100183)
Supplement: Supplementary file 1 [file mmc1.docx]

**Supplementary Materials**

**TCA Cycle Intermediates as an Adjunct Strategy for Human iPSC-Derived Cardiomyocyte Maturation**

**Keshav Narayan Alagarsamy^a,^ Emilee Bueckert^a^, Mehak Gupta^a^, Michel Aliani^b^, Sanjiv Dhingra^a,*^**

^a^Institute of Cardiovascular Sciences, St. Boniface Hospital Albrechtsen Research Centre, Department of Physiology and Pathophysiology, Max Rady College of Medicine, Rady Faculty of Health Sciences, University of Manitoba, Winnipeg, Manitoba, R2H 2A6, Canada

^b^Division of Neurodegenerative Disorders, St. Boniface General Hospital Albrechtsen Research Centre, University of Manitoba, Winnipeg, Canada.

***Correspondence:**

**Sanjiv Dhingra, PhD, FAHA, FIACS, FAPS**

Professor

Institute of Cardiovascular Sciences

St. Boniface Hospital Albrechtsen Research Centre

Department of Physiology and Pathophysiology

Rady Faculty of Health Sciences, Max Rady College of Medicine

Biomedical Engineering Program

University of Manitoba

R-3028-2, 351 Tache Avenue, Winnipeg, R2H2A6, Canada

Email: [sdhingra@sbrc.ca](mailto:sdhingra@sbrc.ca)


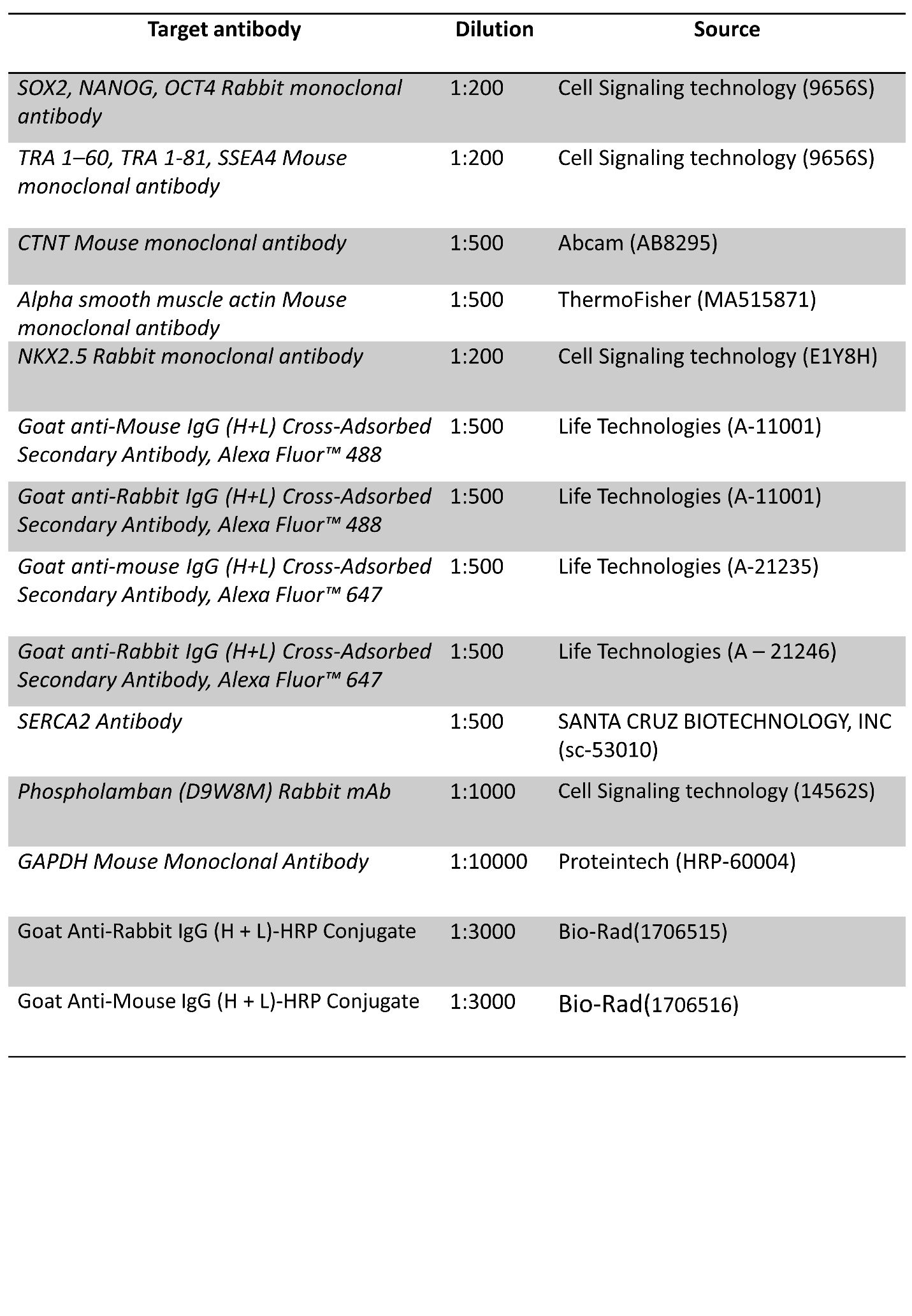


**Table S1:** Table contains list of antibodies and their dilutions used in the experiments for evaluating the pluripotent, cardiac specific markers and calcium handling.


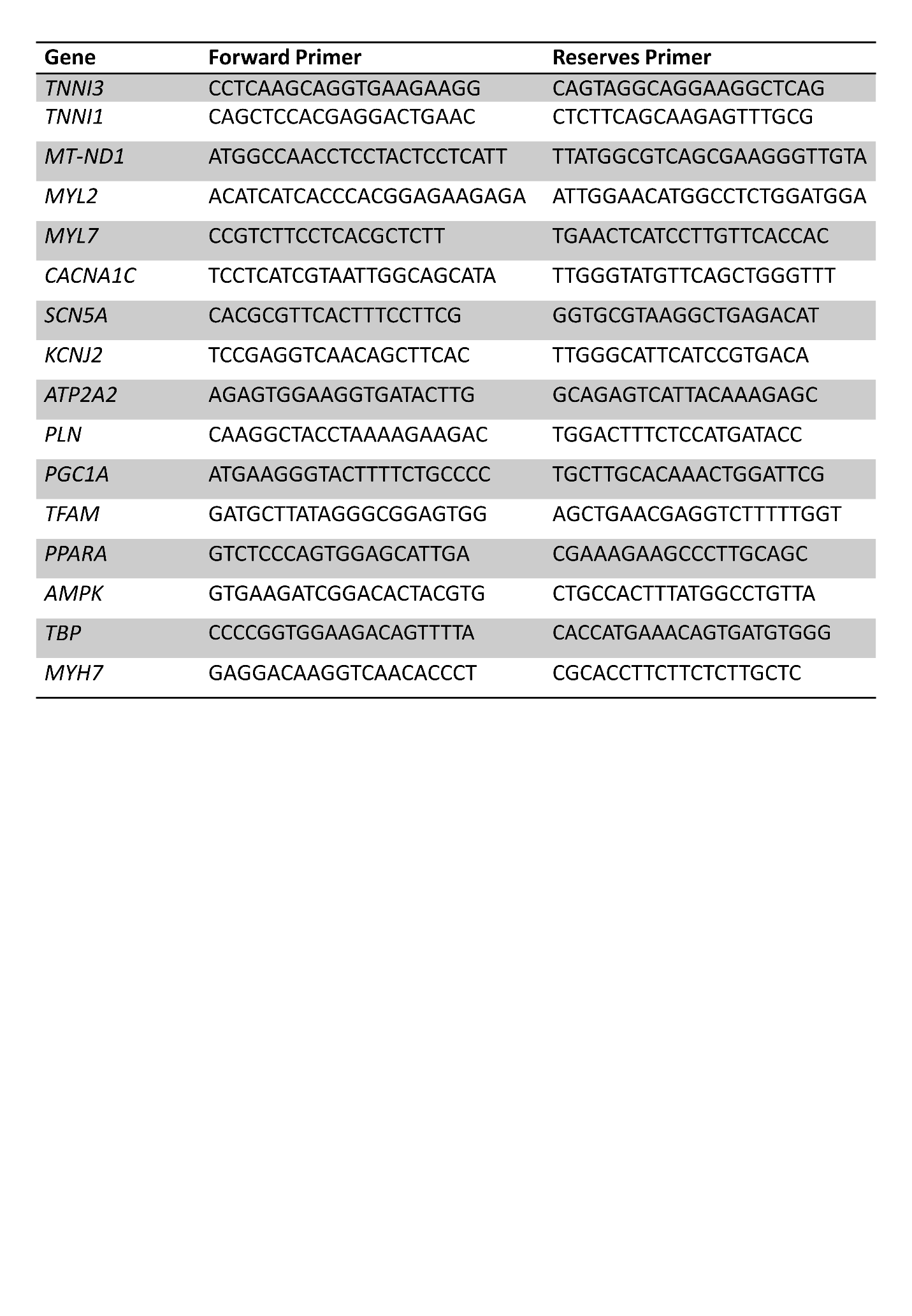


**Table S2:** Table contains the list of genes with forward & reverse primers for evaluating the sarcomere, ion channel, calcium handling and metabolism related genes of iPSC-CM.


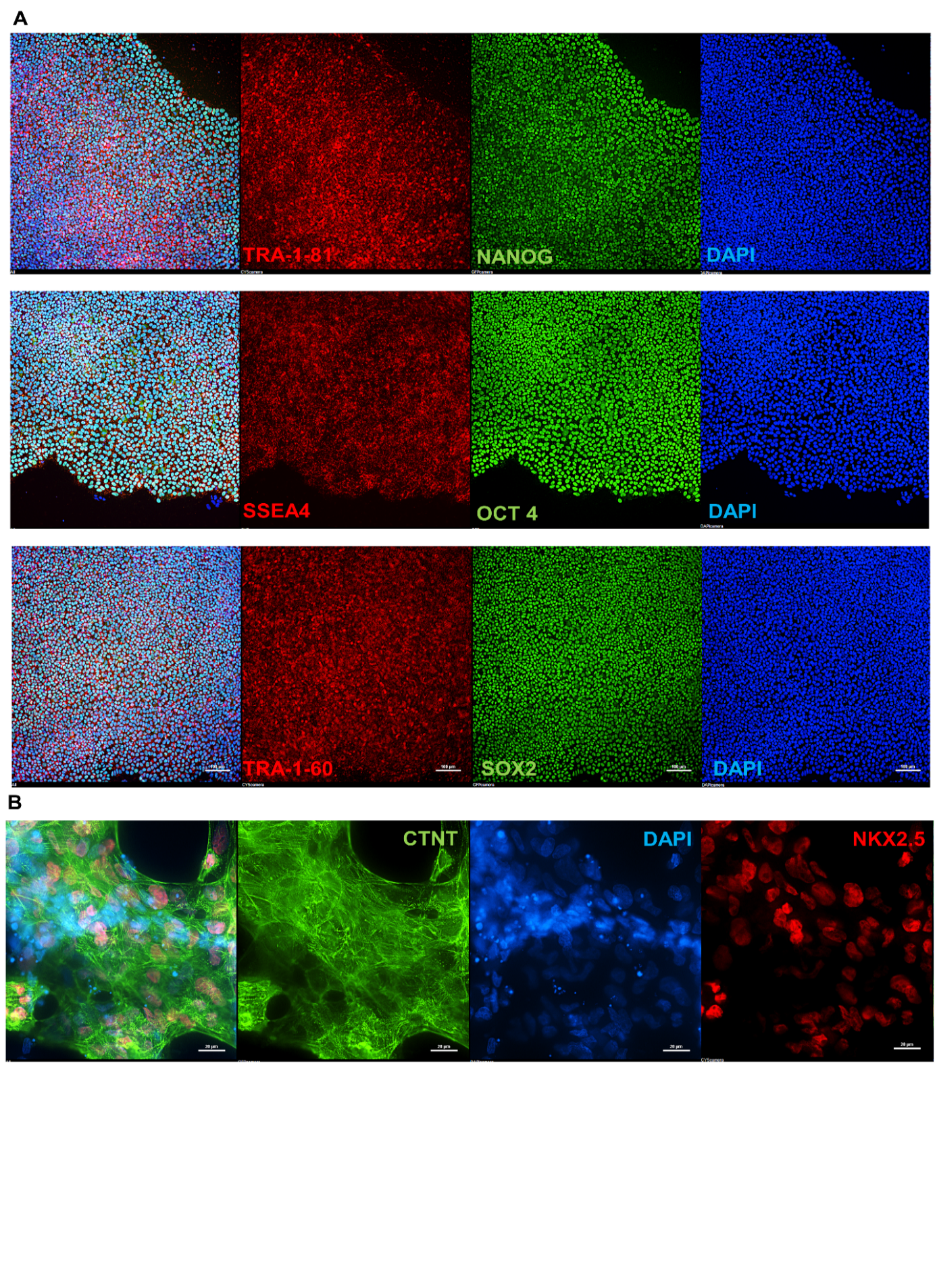


**Figure S3: Characterization of iPSC and iPSC-CM**: A) Representative immunofluorescence images showing the expression of pluripotency markers in iPSC. Nuclear markers SOX2, OCT4, and NANOG are visualized in green, and the surface markers TRA-1-81, TRA-1-60, and SSEA4 are shown in red. B) Characterization of iPSC-CM at day 10 of differentiation using immunofluorescence staining for cardiac-specific markers, cTnT (green) and NKX2.5 (red), with nuclei were stained with DAPI (blue).


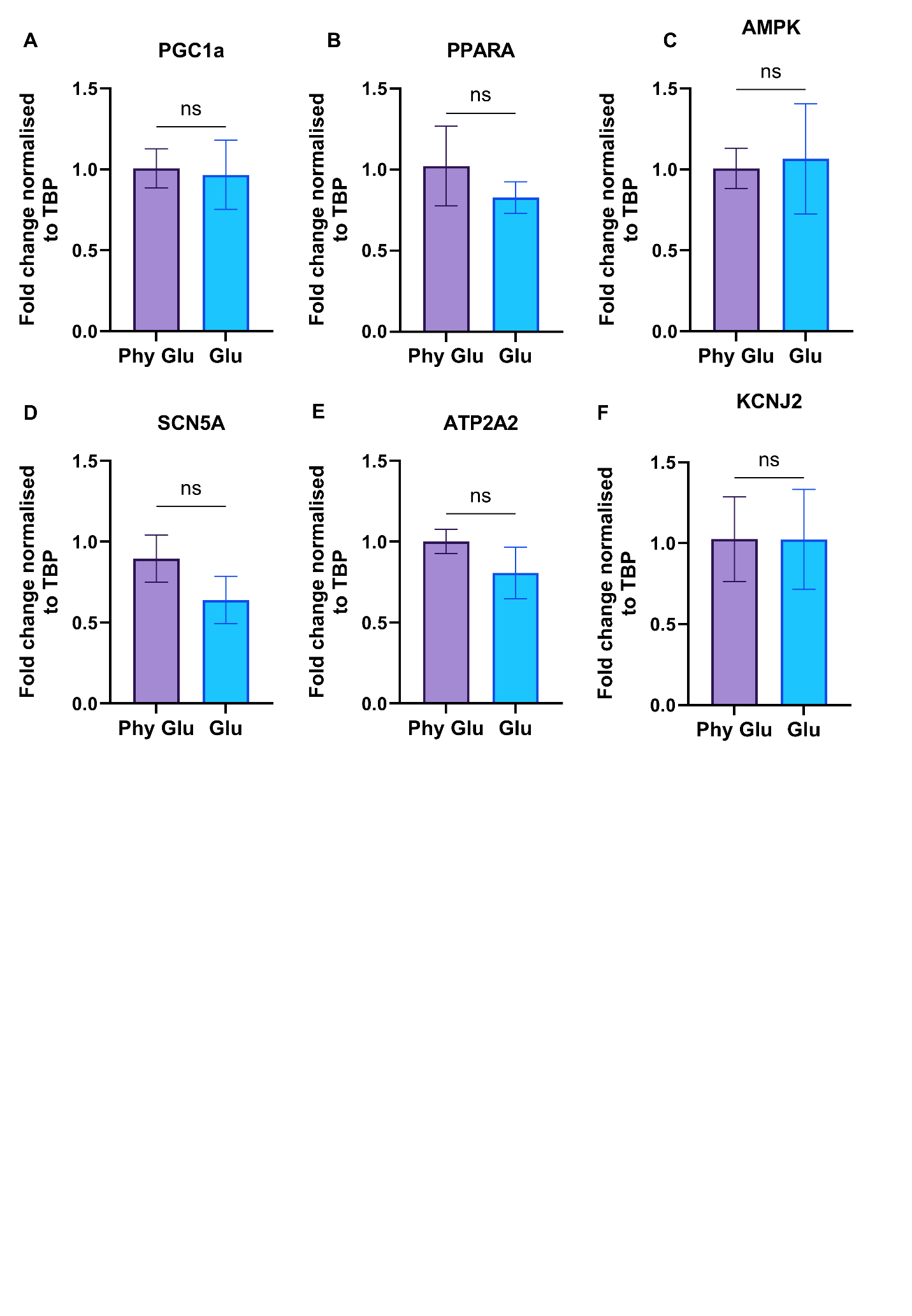


**Figure S4: qPCR analysis of physiological glucose (Phy Glu) and glu rich media (Glu):** A-F) q-PCR analysis of genes associated with metabolism (PGC1a, PPARA and AMPK) and electrophysiology (SCN5A, ATP2A2 and KCNJ2) in iPSC-CM cultured for 7 days in glucose rich (Glu) and physiological glucose (Phy Glu) media (*n* = 3-4 for each group). Relative expression levels were normalized to the housekeeping gene (TBP) and compared across groups. Data are presented as mean ± SD. Statistical significance was assessed using two-tailed T-test.
